# Changes in physical activity and sedentary behavior during the first COVID-19 pandemic- restrictions in Germany: a nationwide survey: Running head: physical activity during the COVID-19 restrictions

**DOI:** 10.1186/s12889-024-17675-y

**Published:** 2024-02-12

**Authors:** Florian Herbolsheimer, Annette Peters, Sarah Wagner, Stefan N. Willich, Lilian Krist, Tobias Pischon, Katharina Nimptsch, Sylvia Gastell, Mirko Brandes, Berit Brandes, Tamara Schikowski, Börge Schmidt, Karin B. Michels, Rafael Mikolajczyk, Volker Harth, Nadia Obi, Stefanie Castell, Jana K. Heise, Wolfgang Lieb, Katrin Franzpötter, André Karch, Henning Teismann, Henry Völzke, Claudia Meinke-Franze, Michael Leitzmann, Michael J. Stein, Hermann Brenner, Bernd Holleczek, Andrea Weber, Barbara Bohn, Alexander Kluttig, Karen Steindorf

**Affiliations:** 1 Division of Physical Activity, Prevention and Cancer, German Cancer Research Center (DKFZ), Heidelberg, Germany; 2 Institute of Epidemiology, Helmholtz Zentrum München – German Research Center for Environmental Health (GmbH), Neuherberg, Germany; 3 Institute of Social Medicine, Epidemiology and Health Economics, Charité – Universitätsmedizin Berlin, Berlin, Germany; 4 Molecular Epidemiology Research Group, Max Delbrück Center for Molecular Medicine in the Helmholtz Association (MDC), Berlin, Germany; 5 German Institute of Human Nutrition Potsdam Rehbruecke, Nuthetal, Germany; 6 Leibniz Institute for Prevention Research and Epidemiology – BIPS, Bremen, Germany; 7 IUF – Leibniz Research Institute for Environmental Medicine, Duesseldorf, Germany; 8 Institute for Medical Informatics, Biometry and Epidemiology, Essen University Hospital, Essen, Germany; 9 Institute for Prevention and Cancer Epidemiology, Faculty of Medicine and Medical Center, University of Freiburg, Freiburg, Germany; 10 Institute for Medical Epidemiology, Biometrics, and Informatics, Interdisciplinary Center for Health Sciences , Medical Faculty of the Martin-Luther University Halle-Wittenberg, Halle, Germany; 11 Institute for Occupational and Maritime Medicine Hamburg (ZfAM), University Medical Centre Hamburg-Eppendorf (UKE), Hamburg, Germany; 12 Helmholtz Centre for Infection Research, Brunswick, Germany; 13 Institute of Epidemiology, University of Kiel, Kiel, Germany; 14 Institute of Epidemiology and Social Medicine, University of Münster, Münster, Germany; 15 Institute for Community Medicine, University Medicine Greifswald, Greifswald, Germany; 16 University of Regensburg, Regensburg, Germany; 17 Division of Clinical Epidemiology and Aging Research, German Cancer Research Center (DKFZ), Heidelberg, Germany; 18 Saarland Cancer Registry, Saarbrücken, Germany; 19 NAKO e.V., Heidelberg, Germany

**Keywords:** COVID-19, Physical activity, Sedentary behavior, Lockdown, Pandemic restrictions, Depression

## Abstract

**Background:**

The COVID-19 pandemic restrictions posed challenges to maintaining healthy lifestyles and physical well-being. During the first mobility restrictions from March to mid-July 2020, the German population was advised to stay home, except for work, exercise, and essential shopping. Our objective was to comprehensively assess the impact of these restrictions on changes in physical activity and sedentary behavior to identify the most affected groups.

**Methods:**

Between April 30, 2020, and May 12, 2020, we distributed a COVID-19-specific questionnaire to participants of the German National Cohort (NAKO). This questionnaire gathered information about participants’ physical activity and sedentary behavior currently compared to the time before the restrictions. We integrated this new data with existing information on anxiety, depressive symptoms, and physical activity. The analyses focused on sociodemographic factors, social relationships, physical health, and working conditions.

**Results:**

Out of 152,421 respondents, a significant proportion reported altered physical activity and sedentary behavioral patterns due to COVID-19 restrictions. Over a third of the participants initially meeting the WHO’s physical activity recommendation could no longer meet the guidelines during the restrictions. Participants reported substantial declines in sports activities (mean change (*M*) = -0.38; 95% CI: -.390; -.378; range from -2 to + 2) and reduced active transportation (*M* = -0.12; 95% CI: -.126; -.117). However, they also increased recreational physical activities (*M* = 0.12; 95% CI: .117; .126) while engaging in more sedentary behavior (*M* = 0.24; 95% CI: .240; .247) compared to pre-restriction levels. Multivariable linear and log-binomial regression models indicated that younger adults were more affected by the restrictions than older adults. The shift to remote work, self-rated health, and depressive symptoms were the factors most strongly associated with changes in all physical activity domains, including sedentary behavior, and the likelihood to continue following the physical activity guidelines.

**Conclusions:**

Mobility patterns shifted towards inactivity or low-intensity activities during the nationwide restrictions in the spring of 2020, potentially leading to considerable and lasting health risks.

**Supplementary Information:**

The online version contains supplementary material available at 10.1186/s12889-024-17675-y.

## Introduction

In response to the COVID-19 pandemic, governments worldwide implemented various forms of lockdown to mitigate the spread of SARS-CoV-2. However, these measures also raised concerns about the potentially negative effects of physical inactivity resulting from such restrictions [1]. On March 22nd, 2020, the German government introduced a series of prevention and control measures to curb the spread of the virus. These measures included closing schools and restaurants, nationwide contact restrictions, travel warnings, and home quarantine [2].

Unavoidably, restricted travel and outdoor activity disrupted people’s daily routines and daily activities, leading to reduced levels of physical activity, along with increased sedentary behavior and more screen time. Nevertheless, scholars emphasized the importance of sustaining physical activity at home to maintain good health and bolster the immune system function in this challenging environment [3, 4]. Regular physical activity has been linked to improved immune function, respiratory health, and a reduction in systemic inflammation, potentially offering protection against infections like COVID-19 [5].

Recent research overviews highlighted a global decline in physical activity levels due to COVID-19-related restrictions [6–8]. National and international ad hoc online surveys have consistently shown a reduced engagement in physical activities and a rise in sedentary behavior [9–12]. Large cohort studies conducted in the UK, France, the US, and Australia corroborate this decline in physical activity during the COVID-19 restrictions [13–16].

Thereby, the ability to remain physically active during the lockdown and mobility restrictions may depend on various factors, including (1) individual characteristics such as age, education, mental and physical health, (2) social factors like working conditions and social support, as well as (3) environmental conditions, as the local COVID-19 incidence rates [17].

Divergent responses across age groups have emerged. Older adults might have exhibited greater caution when leaving their homes due to chronic health conditions, while younger adults might have experienced more disruptions to their daily routines. Changes in working hours and conditions, along with the closure of leisure establishments and childcare facilities, might have impacted the everyday lives of younger adults. In the US, younger individuals have shown reduced physical activity and increased sedentary behavior, with extended periods of sitting and screen time [15]. Conversely, a French cohort revealed that older adults experienced the most decrease in physical activity [14]. In a UK cohort, the youngest and the oldest age groups were mostly affected, while middle-aged individuals were the least affected [16]. Across all age groups, previously physically active individuals experienced a more substantial decline in physical activity than those who were relatively inactive before. Similar patterns have been observed in several studies conducted in Canada, Spain, and Brazil during the COVID-19 restrictions [18–20].

The COVID-19 pandemic has had a wide range of psychological and social effects, with heightened fears of illness and death, along with feelings of helplessness, leading to increased symptoms of depression and anxiety [21, 22]. Therefore, physical activity has been proposed as a remedy to counter the impact of stay-at-home orders on mental health. A recent review indicated that physically active individuals in all age groups reported higher well-being, quality of life, and fewer depressive symptoms, anxiety, and stress [23].

Studies have highlighted a rise in self-reported loneliness amid the COVID-19 mobility restrictions [24, 25]. This increase in loneliness serves as a notable barrier to staying physically active, a challenge that has become especially prominent during the pandemic [26]. During this period, assessments conducted in daily life showed that increased loneliness levels correlated, within individuals, with reduced engagement in moderate-to-vigorous physical activity [27].

Additionally, the shift to working from home might have significantly altered physical activity and sedentary behavior [28]. A systematic review by Wilms et al. found that working from home was related to increased sedentary time and decreased engagement in all dimensions of physical activity [29].

Assessing the impact of the COVID-19 restrictions on physical activity and sedentary behavior in high-risk and vulnerable populations is globally recognized as a critical necessity [30]. The present study utilizes data from the German National Cohort (NAKO Gesundheitsstudie, NAKO) to investigate the pandemic’s impact and the consequences of mobility restrictions on daily physical activity patterns in Germany. Based on the literature, we hypothesized that during mobility restrictions, physical activity decreased and sedentary time increased throughout the German population. Additionally, we anticipate observing distinctly altered physical activity patterns based on participants’ characteristics, social factors, and environmental conditions.

## Methods

### Study population

NAKO is a population-based German cohort study that includes randomly selected participants from the general population. The primary aim of NAKO is to investigate the etiology of major chronic diseases, their preclinical stages, and functional health impairments. The study conducted baseline examinations at 18 regional study centers between 2014 and 2019, enrolling 205,053 participants aged 20 to 69 years. Notably, the study oversampled individuals above 40 years of age [31]. Inclusion criteria encompassed age between 20 and 69 years, proficiency in the German language, and residency in one of the 18 study regions. Informed consent was obtained from all subjects involved in the study. Ethics committees of all 18 study centers approved the NAKO study protocol.

A specific questionnaire tailored to COVID-19 was administered between April 30, 2020, and May 12, 2020, to all 197,834 participants who consented to be contacted again. Participants with a valid e-mail address received the questionnaire electronically through a web-based survey tool. For those without e-mail addresses, a paper questionnaire was sent by mail. Participants self-reported demographic information, information about COVID-19-related restrictions they were adhering to, alterations in COVID-19-related health behaviors, and their mental health status for anxiety and depressive symptoms. Baseline data were utilized to monitor shifts in anxiety and depressive symptoms over time.

### Measurement of physical activity in the COVID questionnaire and the baseline assessment of NAKO

The COVID questionnaire asked two questions about physical activity and sedentary behavior. The initial question focused on changes in physical activity and sedentary behavior due to pandemic-related restrictions, inquiring: “How has your physical activity changed due to the coronavirus pandemic in the following domains?” The following six domains served as separate outcomes: 1) physical activity at work, 2) household activities, 3) recreational activities (e.g., gardening, recreational walking), 4) sports activities (e.g., running, strength training), 5) activities for transport, and 6) sedentary behavior. The response options included “much less than before” (-2), “a little less than before” (-1), “the same” (0), “a little more than before” (1), and “much more than before” (2).

A subsequent question documented the adherence to the WHO physical activity guidelines (i.e., 150 min of moderate-to-vigorous (MVPA) throughout the week). Participants stated the number of days they engaged in moderate-to-vigorous physical activity, posing the query, “How many days per week did you usually participate in physical activity for 30 min or more, causing an increased breathing rate?”. This was assessed separately for the time before and during the COVID-related restrictions. Individuals were categorized into four groups based on their physical activity trajectories considering both time points: (1) “maintenance category” for those adhering to the WHO guidelines at both time points, (2) “non-achieving category” for those falling below recommended physical activity levels at both time points, (3) “increasing category” for those initially not meeting the recommendations but enhancing activity to reach the guidelines during the restrictions, and (4) “decreasing category” for those unable to maintain recommended activity levels.

### Exposure measures

Baseline physical activity was used as an exposure measure to account for the participant’s physical activity levels before the mobility restrictions. It was assessed using the German self-administered version of the Global Physical Activity Questionnaire (GPAQ) [32]. The GPAQ encompassed sedentary behavior and three domains of physical activity: work, transport, and leisure-time physical activity. Participants were queried about their engagement in each activity performed for at least 10 min during a typical week and the daily time allocated to each activity. Additionally, the questionnaire asked separately how much of the work and leisure-time physical activity was performed on a moderated and vigorous level. Vigorous-intense activities induced notably increased breathing, while moderate-intensity activities led to slightly heavier breathing. Physical activity scores were derived by multiplying the frequency and duration of activities. Total physical activity time was calculated from leisure and transport activities. Work-related physical activity was not included in the total score. The GPAQ demonstrated a moderate-to-weak correlation with accelerometry in previous studies, ranging from 0.17 to 0.34 for moderate physical activity and 0.10 to 0.64 for vigorous physical activity [33].

### Mental and physical health

Participants completed the Patient Health Questionnaire-Depression module (PHQ-9) [34] and the General Anxiety Disorder Scale (GAD-7) [35] at baseline and in the COVID-specific questionnaire. The total scores ranged from 0 to 27 and 0 to 21, respectively, with higher scores indicating more depressive symptoms or anxiety levels. Both scales demonstrated high internal consistency, with Cronbach’s α = 0.87 and α = 0.86, respectively. Self-reported physical health was assessed on a 5-point Likert scale ranging from 1 “poor” to 5 “excellent”. Additionally, participants gauged their current health compared to pre-COVID-19 conditions on a 5-point Likert scale spanning “much worse now than before (0)” to “much better now than before (5)”.

### Assessment of working conditions during COVID-19

Working condition changes due to COVID-19 were surveyed. Response options, on a multi-choice scale, encompassed reduced working hours, increased working hours, switched to remote work, and terminated employment.

### Loneliness

Derived from the UCLA Loneliness Scale [36] and translated into German [37], the loneliness scale evaluated participants’ frequency of experiencing loneliness through indicators like missing company, feeling like an outsider, and social isolation. Responses ranged on a 5-point Likert scale from “never” = 0 to “very often” = 4 (range 0 – 12), with higher values denoting greater loneliness. This scale exhibited high internal consistency (Cronbach’s α = 0.73).

### Demographic characteristics

Baseline demographic data encompassed age, sex, highest education level (using the International Standard Classification of Education 97 (ISCED-97)), migration background, sports club membership, and detailed household composition, including the presence of children under nine years old.

### Environmental measures

Existing evidence indicated that heightened incidence rates correlated with restrictions in daily activities [10]. Therefore, regional COVID-19 incident cases were obtained from official registries coinciding with completing the individual COVID-19 questionnaire. When regional incident rates surpassed three times the national mean, they were categorized as exceptionally high. An indicator variable addressed exceptionally high regional incidence rates. Another indicator variable accounted for the late return of questionnaires in June 2020, when COVID-19 regulations had been loosened.

Taken together, we accounted for twenty-one factors, including six participant characteristics, four social measures, two environmental variables, four work-related information, and five aspects of physical and mental health.

### Statistical analysis

Out of 197,834 initially contacted participants, 160,252 completed the COVID-19 questionnaires. Exclusions included 7,831 respondents returning their questionnaire after June 30, 2020, implausible physical activity patterns (simultaneous sharp increase in all activities and sedentary time), or missing information on all physical activity measures (Supplement 1). Missing data analysis indicated comparability to the final analytic sample of 152,421 individuals regarding mean age, health status or depressive symptoms, and baseline physical activity. Missing data from exposure measures were replaced using multiple imputations (Supplement 2). Multiple imputations generated ten datasets using chained equations. Continuous data were imputed with linear regression. For categorical measures, we applied ordinal logistic regression. Estimates from these datasets were pooled using Rubin’s rules [38].

Descriptive statistics summarized the analytic study sample of 152,421 individuals, presenting means and standard deviations (SDs) for continuous variables and proportions for categorical variables. Log-binomial regression models were fitted for categorized data, exploring shifts in adhering to the 150 min of MVPA per week guideline. Comparisons involved the “decreasing” with “maintenance”, and the “increasing” vs. “never achieving” categories. We examined variations in several physical activity domains and sedentary behavior by applying multivariable linear regression models. All models were adjusted for baseline physical activity, individual characteristics, social influences, and environmental characteristics to pinpoint the most relevant factors linked to self-reported physical activity and sedentary behavior changes during COVID-19 restrictions.

We additionally reported the effect size based on Cohen’s f^2^, which allows an evaluation of local effect size within the context of the multivariate regression model. Values of 0.02, 0.30, and 0.5 denoted small, medium, and large effects, respectively [39]. Lastly, we examined potential effect modification by age. Analyses were conducted using STATA 15.0 (College Station, Texas, USA).

## Results

Table 1 presents NAKO participants’ characteristics. In brief, participants (*N* = 152,421) were, on average, 53.7 years old (median = 55, IQR = 46—64), and 52.1% were female. The majority cohabited with partners, and 10.6 percent lived together with children under nine years old. Over a quarter of respondents reported altered working conditions due to the COVID-19 pandemic or the associated restrictions, with a switch to remote work (28.1%) as the most frequent answer.

**Table 1:** Characteristics of the study sample (*N* = 152,421)

	Mean (SD)/ Percentage	
**Socio-demographics**^**a**^		
Age (mean(SD))	53.7	(12.8)
Female (%)	52.1	
Education (%)		
Primary education	1.7	
Secondary education	36.9	
Academic education	53.1	
Unknown	8.3	
Migration background (%)	14.1	
Unemployed (%)	2.0	
**Altered working condition (%)**^**b**^		
Working hours reduction	17.1	
Working hours increase	7.3	
Remote work	28.1	
Dismissal from the job	0.0	
**Health**		
Self-rated health (mean(SD))^b,c^	3.5	(0.8)
Perceived changes in self-reported health (mean(SD))^b, c^	0.0	(0.0)
Anxiety (mean(SD))^a,d^	3.1	(3.1)
Depression (mean(SD))^a,d^	3.8	(3.6)
Δ Depression (mean(SD))^a,b^	0.3	(3.5)
**Social factors**		
Living with children (%)^b^	10.6	
Living alone (%)^b^	18.0	
Loneliness (mean(SD))^b^	1.9	(1.6)
Member of a sports club (%)^a^	40.4	
**Physical activity**		
Physical activity (minutes/ day) (mean(SD))^a^	14.8	(20.8)

*N* = 152,421; education is based on ISCED-97 classification; ^a^ assessed at baseline interview; ^b^ assessed at COVID-19 interview; ^c^ higher values point towards a better health status; ^d^ higher values represent more symptoms; ^e^ change between baseline and COVID-follow-up

**Fig. 1:**
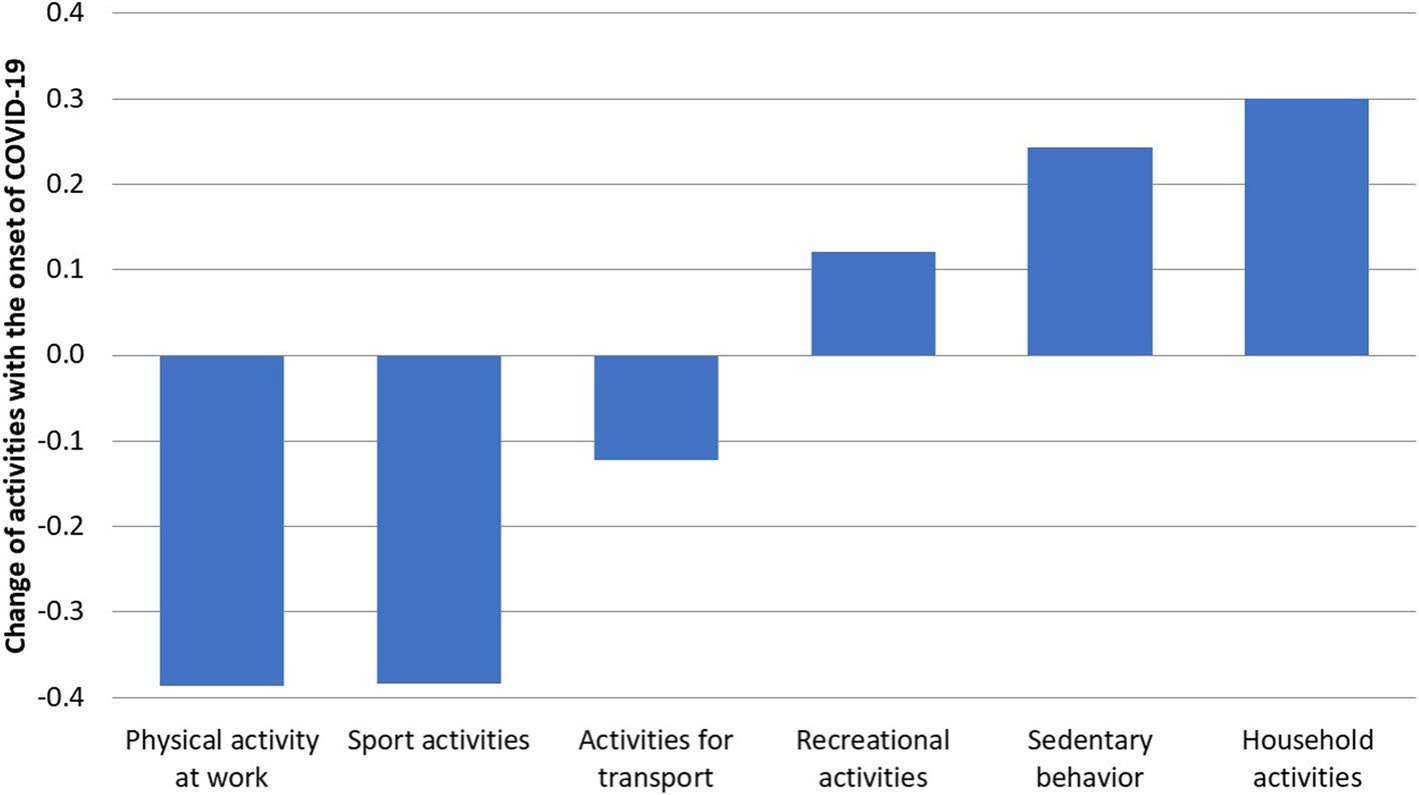
Mean changes in different domains of physical activity on a 5-point Likert Scale ranging from -2 (much less than before) to + 2 (much more than before) ordered by the amount of change

**Fig. 2:**
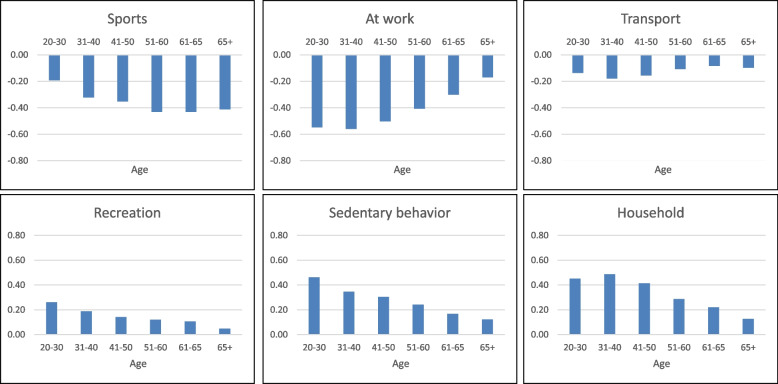
Changes in different age groups by sedentary behavior and domains of physical activity ranging from -2 (much less than before) to + 2 (much more than before)

Regarding the five physical activity domains and sedentary behavior, 32.6% to 54.6% of participants reported modified patterns due to COVID-19-related restrictions (Supplement 3). Participants engaged in fewer days of sufficient MVPA on average (M_Pre-COVID_ = 3.1 days; M_COVID_ = 2.6 days, *p* < 0.001), and 39.4% of those who adhered to the WHO physical activity guidelines before the pandemic fell below the threshold after the onset of the COVID-19 restrictions. Moreover, on the 5-point Likert scale ranging from -2 to + 2, respondents reported fewer sports activities (*M* = -0.38; 95% confidence intervals (CI): -0.390; -0.378), fewer physical activities for transport (*M *= -0.12; 95% CI: -0.126; -0.117), and increased sedentary behavior (*M* = 0.24; 95% CI: 0.240; 0.247). Simultaneously, recreational physical activities (*M* = 0.12; 95% CI: 0.117; 0.126) were performed more often compared to pre-restriction levels (Fig. 1). Notably, the most substantial change in physical activity and sedentary behavior occurred among individuals aged 19 to 40 years (Fig. 2).

Multivariable log-binomial regression analyses were conducted for trajectories aligning with WHO’s physical activity guidelines (Table 2), and multivariate linear regression models were applied for the five physical activity domains and sedentary behavior (Table 3). Working condition shifts, depressive symptoms and physical health were all substantially associated with alterations in physical activity and sedentary behavior (Tables 2 and 3). Those transitioning to remote work reported reduced physical activity at work (β = -0.28; 95% CI: -0.283; -0.274; f^2^ = 0.07) and less time spent on active transport (β = -0.10; 95% CI: -0.101; -0.091; f^2^ = 0.02). But they also reported being concurrently more sedentary (β = 0.19; 95% CI: 0.186; 0.196 f^2^ = 0.03) and were more likely to deviate from activity guidelines (OR = 2.05; 95% CI: 1.94; 2.17) compared to those remaining active.

**Table 2:** Log-binomial regressions for the relationship between participants’ characteristics and meeting the physical activity guidelines of 150 min per week before and after the COVID-19 restrictions

	Increase category					Decrease category				
	(Ref. Never achieving category)					(Ref. Maintain category)				
	OR	*95% CI*				OR	*95% CI*			
Age^b^	1.00	(0.99 1.00)				0.99	(0.99 1.00)			
Female^a^	1.27	(1.21 1.33)				1.10	(1.05 1.15)			
Education^a^										
Primary (Ref.)										
Secondary	1.00	(0.81 1.24)				0.99	(0.82 1.19)			
Tertiary	1.12	(0.90 1.38)				1.03	(0.85 1.24)			
Unknown	1.20	(0.96 1.49)				1.01	(0.83 1.24)			
Migrant background^a^	1.13	(1.06 1.21)				1.37	(1.28 1.45)			
**Altered working condition**^**b**^										
Worktime reduction	1.27	(1.21 1.34)				1.03	(0.97 1.10)			
Worktime increase	0.80	(0.73 0.88)				0.98	(0.90 1.07)			
Remote work	1.36	(1.29 1.43)				2.05	(1.94 2.17)			
Dismissal from the job	2.26	(1.78 2.86)				1.41	(1.05 1.89)			
**Health**										
Self-rated health^b,c^	1.30	(1.26 1.35)				0.93	(0.90 0.96)			
Changes in self-rated health^b,c^	1.98	(1.90 2.05)				0.69	(0.66 0.72)			
Anxiety^a, d^	1.03	(1.02 1.04)				0.98	(0.97 0.99)			
Depression^a,d^	0.99	(0.98 1.00)				1.05	(1.04 1.06)			
Δ depression (T2-T1)^e^	1.00	(1.00 1.01)				1.05	(1.05 1.06)			
**Social factors**										
Household with children^b^	0.71	(0.66 0.77)				1.27	(1.17 1.38)			
Feeling of loneliness^b^	1.07	(1.05 1.08)				1.18	(1.16 1.20)			
Member of a sports club^a^	1.27	(1.21 1.33)				1.44	(1.37 1.50)			
Physical activity^a^	1.01	(1.01 1.01)				0.99	(0.99 1.00)			
N	116,007					36,408				

*N* = 152,421; ^a^ assessed at baseline interview; ^b^ assessed at COVID-19 interview; ^c^ higher values point towards a better health status; ^d^ higher values represent more symptoms; ^e^ change between baseline and COVID-follow-up; additionally adjusted for unemployment, living alone, regional number of covid-cases at time of the interview, month of interview

**Table 3:** Linear regressions for the relationship between participants’ characteristics and increases in time spent in various physical activity domains compared to pre-COVID states

	Sports				At work				Transport				Recreation				Sedentary behavior				Household			
	β	95%-CI			β	95%-CI			β	95%-CI			β	95%-CI			β	95%-CI			β	95%-CI		
Age^b^	-0.05	(-.054 -.042)			0.04	(.037 .049)			0.00	(-.009 .003)			-0.03	(-.033 -.021)			-0.07	(-.076 -.064)			-0.08	(-.081 -.069)		
Female^a^	0.01	(.004 .014)			-0.03	(-.037 -.027)			0.01	(.002 .012)			0.03	(.029 .039)			0.01	(.007 .017)			0.07	(.068 .078)		
Education^a^																								
Primary (Ref.)																								
Secondary	-0.01	(-.034 .005)			0.00	(-.014 .022)			0.01	(-.004 .034)			0.02	(.004 .041)			0.01	(-.005 .033)			0.03	(.001 .044)		
Tertiary	-0.01	(-.025 .013)			-0.03	(-.045 -.008)			0.00	(-.018 .022)			0.05	(.031 .07)			0.05	(.034 .074)			0.08	(.061 .099)		
Unknown	0.00	(-.014 .008)			-0.01	(-.019 .003)			0.00	(-.012 .011)			0.02	(.004 .027)			0.04	(.024 .047)			0.03	(.021 .043)		
Migrant background^a^	-0.02	(-.027 -.018)			-0.02	(-.025 -.015)			-0.03	(-.036 -.026)			-0.05	(-.060 -.05)			0.02	(.014 .024)			0.02	(.013 .023)		
**Altered working condition**^**b**^																								
Worktime reduction	0.02	(.013 .023)			-0.08	(-.080 -.071)			0.04	(.033 .044)			0.06	(.057 .067)			-0.01	(-.016 -.006)			0.07	(.066 .076)		
Worktime increase	-0.03	(-.033 -.023)			0.06	(.053 .062)			0.00	(-.010 .001)			-0.03	(-.039 -.029)			0.02	(.020 .029)			-0.02	(-.024 -.014)		
Remote work	0.03	(-.002 .009)			-0.28	(-.283 -.274)			-0.10	(-.101 -.091)			0.04	(.039 .049)			0.19	(.186 .196)			0.16	(.153 .162)		
Dismissal from the job	0.01	(.001 .011)			-0.03	(-.035 -.025)			0.00	(-.003 .007)			0.01	(.002 .012)			0.01	(.002 .012)			0.02	(.019 .029)		
**Health**																								
Self-rated health^b,c^	0.05	(.041 .053)			0.01	(.004 .015)			0.04	(.032 .044)			0.05	(.043 .054)			0.00	(-.009 .002)			0.03	(.027 .038)		
Changes in self-rated health^b,c^	0.17	(.161 .177)			0.05	(.044 .054)			0.10	(.095 .106)			0.13	(.122 .132)			-0.06	(-.065 -.055)			0.07	(.065 .076)		
Anxiety^a, d^	0.03	(.025 .041)			0.02	(.014 .029)			0.04	(.027 .043)			0.04	(.036 .052)			-0.02	(-.031 -.016)			0.04	(.031 .046)		
Depression^a,d^	-0.07	(-.082 -.064)			-0.08	(-.092 -.076)			-0.09	(-.100 -.082)			-0.10	(-.110 -.092)			0.07	(.060 .077)			-0.05	(-.058 -.042)		
Δ depression (T2-T1)^e^	-0.06	(-.065 -.053)			-0.09	(-.092 -.08)			-0.07	(-.078 -.065)			-0.08	(-.081 -.069)			0.07	(.062 .074)			-0.02	(-.024 -.012)		
**Social factors**																								
Household with children^b^	-0.02	(-.023 -.013)			0.00	(-.007 .003)			-0.02	(-.028 -.017)			-0.01	(-.014 -.004)			-0.04	(-.046 -.035)			0.06	(.056 .067)		
Feeling of loneliness^b^	-0.05	(-.050 -.04)			-0.08	(-.087 -.077)			-0.03	(-.034 -.024)			-0.03	(-.033 -.023)			0.04	(.038 .049)			0.03	(.024 .035)		
Member of a sports club^a^	-0.09	(-.094 -.084)			-0.02	(-.024 -.015)			0.03	(.021 .031)			0.03	(.029 .039)			0.01	(.003 .013)			0.03	(.021 .032)		
Physical activity^a^	0.02	(.017 .027)			0.01	(.007 .016)			0.02	(.018 .028)			0.01	(.007 .017)			-0.01	(-.018 -.009)			0.00	(-.007 .003)		
adj. R^2^		.065				.136				.041				.065				.073				.091		

*N* = 152,421; ^a^ assessed at baseline interview; ^b^ assessed at COVID-19 interview; ^c^ higher values point towards a better health status; ^d^ higher values represent more symptoms; ^e^ change between baseline and COVID-follow-up; all models were additionally adjusted for unemployment, living alone, regional exceptionally high number of covid-cases, month of interview

Better perceived health compared to the pre-COVID-19 restrictions correlated with more sports (β = 0.17; 95% CI: 0.161, 0.177; f^2^ = 0.03) and recreational activities (β = 0.13; 95% CI: 0.122; 0.132; f^2^ = 0.02). Conversely, more depressive symptoms reported at the baseline assessment were related to less recreational activities during the mobility restrictions (β = -0.10.; 95% CI:  -0.110; -0.092; f^2^ = 0.004), particularly if symptoms worsened since baseline. Individuals with depressive symptoms were also more likely to forgo the physical activity guidelines (*OR* = 1.05; 95% CI:  1.04; 1.06) than those remaining physically active.

Further analyses revealed that increased depressive symptoms since baseline assessment had a more pronounced effect on sedentary time, particularly among younger respondents. An interaction effect between age and increased depressive symptoms reached statistical significance for sedentary behavior (β = -0.02; 95% CI: -0.027; -0.017), indicating a heightened impact on younger individuals’ sedentary behavior (Supplement 4).

The models also identified significant negative relationships with educational attainment, loneliness, migration background, and exceptionally high regional COVID-19 incidence rates, though with small effect sizes (Tables 2 and 3). A comparison between questionnaires that were returned in June’s first and second halves showed no differences in the estimators.

## Discussion

The present study, encompassing 152,421 adults across various age groups, provides valuable insight into shifts in physical activity patterns at the onset of the COVID-19 pandemic in Germany. Most participants reported reduced physical activity levels, an increase in sedentary time, and more household chores during the COVID-19 restrictions between April and June 2020. Notably, over a third of the participants no longer met the WHO’s physical activity guidelines. These findings align with previous studies documenting similar alterations in physical activity habits during COVID-19 restrictions [8]. For instance, a UK cohort study recorded a 30% decline in the likelihood of engaging in any physical activity [16]. Similarly, a French study reported that over half of participants decreased their physical activity levels and increased sedentary time [14]. In our study, it is remarkable that 32.5 percent reported more recreational activities and 16.2 percent more sports activities than before the COVID-19-related restrictions restrained their activity. Also, six percent initiated efforts to meet the physical activity guidelines despite the challenging conditions of being physically active.

Contrary to our expectations, our study did not find evidence of a substantial decline in physical activity among older adults who, due to their heightened vulnerability, may have been more cautious and restricted their daily activities. Conversely, young adults reported the most significant negative changes across all physical activity domains and sedentary behavior. Specifically, younger adults experienced a notable increase in sedentary time and household activities, coupled with a reduction in work-related activities and time spent on transport. No distinct age-related pattern was observed for sports activities. These findings are consistent with other studies, such as that by Bu et al. [40], which found decreasing physical activity levels in younger adults in England, and smartphone tracking studies in Canada that reported similar results [18]. The more pronounced decline in physical activity among younger adults may be attributed to their higher pre-COVID-19 activity levels, which then leveled off to basal activity levels when restrictions were imposed [41].

Depressive symptoms also played a crucial role when it came to sedentary behavior. Our study revealed that individuals experiencing current or increasing depressive symptoms tended to be more sedentary. This aligns with previous research on the association between depressive symptoms and physical activity [15, 42] or sedentary behavior [43, 44] during the COVID-19 restrictions. Specifically, younger adults (aged between 19 and 49) showed a notable increase in depressive symptoms compared to older adults [21, 45]. Their physical activity and sedentary behavior were more strongly associated with depressive symptoms. These findings align with studies indicating that the COVID-19 restrictions were particularly stressful for younger adults (< 35 years) [46]. Furthermore, a recent meta-analysis highlighted that older adults were the most sedentary group but were also the least affected by the COVID-19 restrictions [47].

Changes in working conditions significantly predicted altered physical activity and sedentary behavior. Our study underscored the bidirectional effects of remote work on physical activity and sedentary behavior. While remote work allowed for more recreational activities, it was also associated with substantially increasing sedentary time. The switch to remote work was simultaneously linked to a higher likelihood of initiating physical activity but also to a higher probability of no longer meeting the physical activity guidelines. These findings align with studies reporting that individuals working remotely were less affected by the restrictions concerning leisure time and physical activity [29, 48]. Additionally, unemployment and student status were associated with the highest odds of reductions in any activities [16]. In summary, altered working conditions significantly impacted the working adult population, as working time is one of the major structuring forces in daily routines.

The COVID-19 pandemic circumstances heightened social isolation and feelings of loneliness. Contrary to our expectations, we found only weak associations between loneliness and the various physical activity outcomes. Similarly, other studies failed to detect a significant relationship between loneliness and physical activity on the between-person level, while within-person patterns were observed [27, 49]. One explanation could be that the mobility restriction put many individuals in similar situations, limiting variability between persons.

Regarding educational levels, our analyses did not indicate that lower-educated individuals were more affected by the COVID-19 restrictions in Germany. Only a slight trend suggested that the highly educated engaged more in recreational and household activities than those with primary education. Additionally, there was almost no relationship between physical activity and having a migration background. These findings parallel those of other international cohort studies, which either reported no effect [14] or indicated a slight relationship between education and physical activity [16]. It is plausible that education was indirectly related to altered physical activity and sedentary time through its association with remote work opportunities, job loss, financial capabilities, and mental resilience to cope with COVID-19-related restrictions.

Our findings strongly advocate promoting physical activity while limiting screen time during the pandemic. Physical activity aids immune health, reduces respiratory illness risk, and serves as a crucial coping strategy to mitigate the impact of social distancing on mental health and well-being. Prior research in Germans indicated an overall decline in all physical activity domains and decreased compliance with aerobic or muscle-strengthening recommendations during the COVID-19 pandemic [48]. Nevertheless, the mobility restrictions might have also enabled opportunities for some groups to be more physically active. A considerable proportion of previously inactive individuals demonstrated increased physical activity during the COVID-19 restrictions, which has also been reported in other studies [14].

The strengths of the current study include its sizeable nationwide sample and its assessment of the physical activity and sedentary behavior of Germans before and during the COVID-19 pandemic. Participants were reached at the early stages of the pandemic in Spring 2020 by online questionnaires or paper–pencil questionnaires, ensuring the inclusion of persons with and without online access. The longitudinal study design, observing the same individuals both before and during the pandemic, provides more robust insights compared to previous studies that mainly relied on one-time assessments during the pandemic or targeted convenient and easily accessible groups, such as students.

However, several limitations should be acknowledged. Firstly, the severe confinement restrictions in Germany lasted from March 21 until June 13 and consequently did not entirely overlap with the study period. Nonetheless, weekend restrictions continued to limit physical activity beyond June 13. Sensitivity analyses comparing June’s first and second halves did not reveal any differences in the estimators. Secondly, physical activity was self-reported and included a recall of pre-COVID-19 activity, which may have been subject to misreporting. Third, this study did not investigate the composition of screen time, which likely incorporated greater exposure to “negative” news that may influence mental health. Lastly, the analyses did not cover environmental factors such as outdoor sports facilities, green areas, and the walkability of the neighborhood, which might have played an additional role in promoting physical activity during the mobility restrictions. Future research should explore the long-lasting effect of COVID-19 restrictions on prospective physical activity and sedentary behavior. Additionally, there is evidence that increases in physical activity were not maintained prospectively while barriers to physical activity persisted [50].

## Conclusion 

In conclusion, COVID-19-related public health measures led to decreased sports activities and a more sedentary lifestyle in Germany. The switch to remote work and (increasing) depressive symptoms showed the most substantial association with changes in physical activity and sedentary behavior. The current findings support the need to monitor physical activity and sedentary behavior in the future and to implement and support measures that promote physical activity, especially in groups most affected by measures like the COVID-19 lockdown.

### Supplementary Information

**Additional file 1:** **Supplement 1.** Flow diagram of the NAKO cohort study. **Supplement 2.** Frequency of missing data. **Supplement 3.** Changes in sedentary behavior and physical activity domains. **Supplement 4.** Interaction between age and the development of depressive symptoms on the change of physical activity and sedentary behavior at the onset of the COVID-19 restrictions.

## Data Availability

Restrictions apply to the availability of these data, which were used under license for the current research and are not publicly available. Data are, however, available via a standardized data usage application procedure (https://transfer.nako.de/transfer/index).

## References

[1] Füzéki E, Groneberg DA, Banzer W (2020). Physical activity during COVID-19 induced lockdown: recommendations. J Occup Med Toxicol.

[2] Die Bundesregierung. 22. März 2020: Regeln zum Corona-Virus. Die Bundesregierung informiert | Startseite. 2020. https://www.bundesregierung.de/breg-de/leichte-sprache/22-maerz-2020-regeln-zum-corona-virus-1733310. Accessed 22 Feb 2023.

[3] Chen P, Mao L, Nassis GP, Harmer P, Ainsworth BE, Li F (2020). Coronavirus disease (COVID-19): The need to maintain regular physical activity while taking precautions. J Sport Health Sci.

[4] Hammami A, Harrabi B, Mohr M, Krustrup P (2022). Physical activity and coronavirus disease 2019 (COVID-19): specific recommendations for home-based physical training. Manag Sport Leis..

[5] Nieman DC (2020). Coronavirus disease-2019: A tocsin to our aging, unfit, corpulent, and immunodeficient society. J Sport Health Sci.

[6] Oliveira MR, Sudati IP, Konzen VDM, de Campos AC, Wibelinger LM, Correa C (2022). Covid-19 and the impact on the physical activity level of elderly people: A systematic review. Exp Gerontol.

[7] Stockwell S, Trott M, Tully M, Shin J, Barnett Y, Butler L (2021). Changes in physical activity and sedentary behaviours from before to during the COVID-19 pandemic lockdown: a systematic review. BMJ Open Sport Exerc Med.

[8] Wunsch K, Kienberger K, Niessner C (2022). Changes in physical activity patterns due to the Covid-19 pandemic: a systematic review and meta-analysis. Int J Environ Res Public Health.

[9] Ammar A, Brach M, Trabelsi K, Chtourou H, Boukhris O, Masmoudi L (2020). Effects of COVID-19 home confinement on eating behaviour and physical activity: results of the ECLB-COVID19 International Online Survey. Nutrients.

[10] Qin F, Song Y, Nassis GP, Zhao L, Dong Y, Zhao C (2020). Physical activity, screen time, and emotional well-being during the 2019 novel coronavirus outbreak in China. Int J Environ Res Public Health.

[11] Rogers NT, Waterlow NR, Brindle H, Enria L, Eggo RM, Lees S (2020). Behavioral change towards reduced intensity physical activity is disproportionately prevalent among adults with serious health issues or self-perception of high risk during the UK COVID-19 lockdown. Front Public Health.

[12] Swain P, James E, Laws JM, Strongman C, Haw S, Barry G (2023). COVID-19: self-reported reductions in physical activity and increases in sedentary behaviour during the first national lockdown in the United Kingdom. Sport Sci Health.

[13] Arundell L, Salmon J, Timperio A, Sahlqvist S, Uddin R, Veitch J (2022). Physical activity and active recreation before and during COVID-19: The Our Life at Home study. J Sci Med Sport.

[14] Charreire H, Verdot C, de SzaboEdelenyi F, Deschasaux-Tanguy M, Srour B, Druesne-Pecollo N (2022). Correlates of changes in physical activity and sedentary behaviors during the COVID-19 lockdown in France: The NutriNet-Santé Cohort Study. Int J Environ Res Public Health..

[15] Meyer J, McDowell C, Lansing J, Brower C, Smith L, Tully M (2020). Changes in physical activity and sedentary behavior in response to COVID-19 and their associations with mental health in 3052 US adults. Int J Environ Res Public Health.

[16] Strain T, Sharp SJ, Spiers A, Price H, Williams C, Fraser C (2022). Population level physical activity before and during the first national COVID-19 lockdown: A nationally representative repeat cross-sectional study of 5 years of Active Lives data in England. Lancet Reg Health Eur.

[17] Sallis JF, Owen N, Glanz K, Rimer BK, Lewis FM (2002). Ecological models of health behavior. Health Behavior and Health Education: Theory, Research, and Practice.

[18] Lesser IA, Nienhuis CP (2020). The impact of COVID-19 on physical activity behavior and well-being of Canadians. Int J Environ Res Public Health.

[19] López-Bueno R, Calatayud J, Andersen LL, Balsalobre-Fernández C, Casaña J, Casajús JA (2020). Immediate impact of the COVID-19 confinement on physical activity Levels in Spanish adults. Sustainability.

[20] Schuch FB, Bulzing RA, Meyer J, López-Sánchez GF, Grabovac I, Willeit P (2022). Moderate to vigorous physical activity and sedentary behavior changes in self-isolating adults during the COVID-19 pandemic in Brazil: a cross-sectional survey exploring correlates. Sport Sci Health.

[21] Peters A, Rospleszcz S, Greiser KH, Dallavalle M, Berger K (2020). The impact of the COVID-19 pandemic on self-reported health: early evidence from the German National Cohort. Dtsch Arztebl Int..

[22] Salari N, Hosseinian-Far A, Jalali R, Vaisi-Raygani A, Rasoulpoor S, Mohammadi M (2020). Prevalence of stress, anxiety, depression among the general population during the COVID-19 pandemic: a systematic review and meta-analysis. Glob Health.

[23] Marconcin P, Werneck AO, Peralta M, Ihle A, Gouveia ÉR, Ferrari G (2022). The association between physical activity and mental health during the first year of the COVID-19 pandemic: a systematic review. BMC Public Health.

[24] O’Sullivan R, Burns A, Leavey G, Leroi I, Burholt V, Lubben J (2021). Impact of the COVID-19 pandemic on loneliness and social isolation: a multi-country study. Int J Environ Res Public Health.

[25] Ernst M, Niederer D, Werner AM, Czaja SJ, Mikton C, Ong AD (2022). Loneliness before and during the COVID-19 pandemic: A systematic review with meta-analysis. Am Psychol.

[26] Holt-Lunstad J, Perissinotto CM (2022). Isolation in the time of Covid: What is the true cost, and how will we know?. Am J Health Promot.

[27] Broen T, Choi Y, Zambrano Garza E, Pauly T, Gerstorf D, Hoppmann CA (2023). Time-varying associations between loneliness and physical activity: Evidence from repeated daily life assessments in an adult lifespan sample. Front psychol..

[28] Wallmann-Sperlich B, Bucksch J, Lendt C, Biallas B, Bipp T, Froboese I. Home office shift and sedentary behaviour in Germany during the COVID-19 pandemic: descriptives and related socioecological correlates. Ergonomics. 2023;:1–12. Online ahead of print. 10.1080/00140139.2023.2202841 37125437

[29] Wilms P, Schröder J, Reer R, Scheit L (2022). The impact of “home office” work on physical activity and sedentary behavior during the COVID-19 pandemic: a systematic review. Int J Environ Res Public Health.

[30] Sallis JF, Adlakha D, Oyeyemi A, Salvo D (2020). An international physical activity and public health research agenda to inform coronavirus disease-2019 policies and practices. J Sport Health Sci.

[31] Peters A, Peters A, Greiser KH, Göttlicher S, Ahrens W, Albrecht M (2022). Framework and baseline examination of the German National Cohort (NAKO). Eur J Epidemiol.

[32] Armstrong T, Bull F (2006). Development of the World Health Organization Global Physical Activity Questionnaire (GPAQ). J Public Health.

[33] Sember V, Meh K, Sorić M, Starc G, Rocha P, Jurak G (2020). Validity and reliability of International Physical Activity Questionnaires for adults across EU countries: systematic review and meta analysis. Int J Environ Res Public Health.

[34] Kroenke K, Spitzer RL, Williams JBW (2001). The PHQ-9: validity ofa brief depression severity measure. J Gen Intern Med.

[35] Spitzer RL, Kroenke K, Williams JBW, Löwe B (2006). A brief measure for assessing generalized anxiety disorder: the GAD-7. Arch Intern Med.

[36] Russell DW (1996). UCLA Loneliness Scale (Version 3): Reliability, validity, and factor structure. J Pers Assess.

[37] Luhmann M, Hawkley LC (2016). Age differences in loneliness from late adolescence to oldest old age. Dev Psychol.

[38] White IR, Royston P, Wood AM (2011). Multiple imputation using chained equations: Issues and guidance for practice. Stat Med.

[39] Cohen J (1992). A power primer. Psychol Bull.

[40] Bu F, Bone JK, Mitchell JJ, Steptoe A, Fancourt D (2021). Longitudinal changes in physical activity during and after the first national lockdown due to the COVID-19 pandemic in England. Sci Rep.

[41] Hino K, Asami Y (2021). Change in walking steps and association with built environments during the COVID-19 state of emergency: A longitudinal comparison with the first half of 2019 in Yokohama, Japan. Health Place.

[42] Bonnell LN, Clifton J, Wingood M, Gell N, Littenberg B (2022). The Relationship Between Mental and Physical Health and Walking During the COVID-19 Pandemic. J Am Board Fam Med.

[43] Bird JM, Karageorghis CI, Hamer M (2021). Relationships among behavioural regulations, physical activity, and mental health pre- and during COVID–19 UK lockdown. Psychol Sport Exerc.

[44] Cheval B, Sivaramakrishnan H, Maltagliati S, Fessler L, Forestier C, Sarrazin P (2021). Relationships between changes in self-reported physical activity, sedentary behaviour and health during the coronavirus (COVID-19) pandemic in France and Switzerland. J Sports Sci.

[45] Berger K, Riedel-Heller S, Pabst A, Rietschel M, Richter D, Lieb W (2021). Einsamkeit während der ersten Welle der SARS-CoV-2-Pandemie – Ergebnisse der NAKO-Gesundheitsstudie. Bundesgesundheitsbl.

[46] Pieh C, Budimir S, Probst T (2020). The effect of age, gender, income, work, and physical activity on mental health during coronavirus disease (COVID-19) lockdown in Austria. J Psychosom Res.

[47] Runacres A, Mackintosh KA, Knight RL, Sheeran L, Thatcher R, Shelley J (2021). Impact of the COVID-19 pandemic on sedentary time and behaviour in children and adults: A systematic review and meta-analysis. Int J Environ Res Public Health.

[48] Füzéki E, Schröder J, Groneberg DA, Banzer W (2021). Physical activity and its related factors during the first COVID-19 lockdown in Germany. Sustainability.

[49] Guerra-Balic M, González-González CS, Sansano-Nadal O, López-Dóriga A, Chin M-K, Ding K (2023). Impact of COVID-19 lockdown on physical activity, insomnia, and loneliness among Spanish women and men. Sci Rep.

[50] Barrett EM, Wyse J, Forde C (2022). Did physical activity and associated barriers change during COVID-19 restrictions in Ireland? Repeated cross-sectional study. Health Promot Int..

