# Supplementary material for: Changes in physical activity and sedentary behavior during the first COVID-19 pandemic- restrictions in Germany: a nationwide survey: Running head: physical activity during the COVID-19 restrictions
Source: BMC Public Health. 2024 Feb 12;24:433. doi: 10.1186/s12889-024-17675-y (PMC10860251; doi:10.1186/s12889-024-17675-y)
Supplement: Supplementary file 1 — Additional file 1: Supplement 1. Flow diagram of the NAKO cohort study. Supplement 2. Frequency of missing data. Supplement 3. Changes in sedentary behavior and physical activity domains. Supplement 4. Interaction between age and the development of depressive symptoms on the change of physical activity and sedentary behavior at the onset of the COVID-19 restrictions. [file 12889_2024_17675_MOESM1_ESM.docx]

**Supplements**

**Supplement 1. Flow diagram of the NAKO cohort study**

**
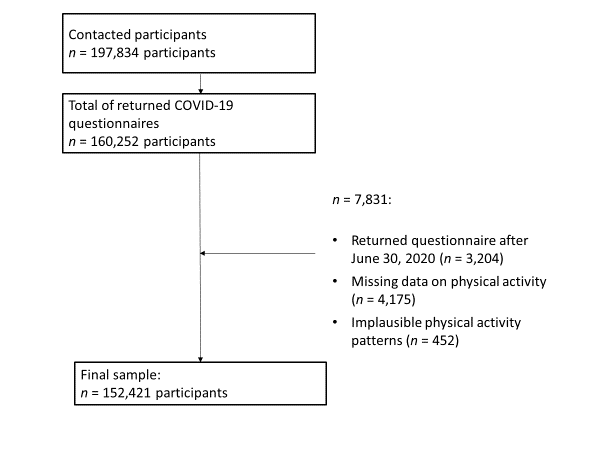
**

**Supplement 2. Frequency of missing data**

|  |  |  |  |  |  |
| --- | --- | --- | --- | --- | --- |
|  | Missing | |  | Complete | |
|  | n | % |  | n | % |
| Self-reported PA change scores (COVID-19 survey) | 0 | 0 |  | 152,421 | 100 |
| Following PA guidelines (COVID-19 survey) |  |  |  |  |  |
| Before COVID-19 restrictions | 4,436 | 2.9 |  | 147,985 | 97.1 |
| With the onset of the restrictions | 5,385 | 3.5 |  | 147,036 | 96.5 |
| GPAQ (baseline survey) | 1,969 | 1.3 |  | 150,452 | 98.7 |
| Age (COVID-19 survey) | 9 | 0.0 |  | 152,412 | 100.0 |
| Sex (baseline survey) | 4 | 0.0 |  | 152,408 | 100.0 |
| Education (baseline survey) | 9 | 0.0 |  | 152,412 | 100.0 |
| Migrant background (baseline survey) | 9 | 0.0 |  | 152,412 | 100.0 |
| Altered working condition (COVID-19 survey) | 9 | 0.0 |  | 152,412 | 100.0 |
| Self-rated health (COVID-19 survey) | 736 | 0.5 |  | 151,685 | 99.5 |
| Changes in self-rated health (COVID-19 survey) | 716 | 0.5 |  | 151,705 | 99.5 |
| Anxiety (COVID-19 survey) | 492 | 0.3 |  | 151,929 | 99.7 |
| Depression (baseline survey) | 549 | 0.4 |  | 151,872 | 99.6 |
| Depression (COVID-19 survey) | 549 | 0.4 |  | 151,872 | 99.6 |
| Household with children (COVID-19 survey) | 9 | 0.0 |  | 152,412 | 100.0 |
| Feeling of loneliness (COVID-19 survey) | 352 | 0.2 |  | 152,069 | 99.8 |
| Member of a sports club (COVID-19 survey) | 9 | 0.0 |  | 152,412 | 100.0 |
| Living alone | 9 | 0.0 |  | 152,412 | 100.0 |
| Notes. n = number of respondents; GPAQ= Global physical activity questionnaire; | | | | | |

**Supplement 3. Changes in sedentary behavior and physical activity domains**


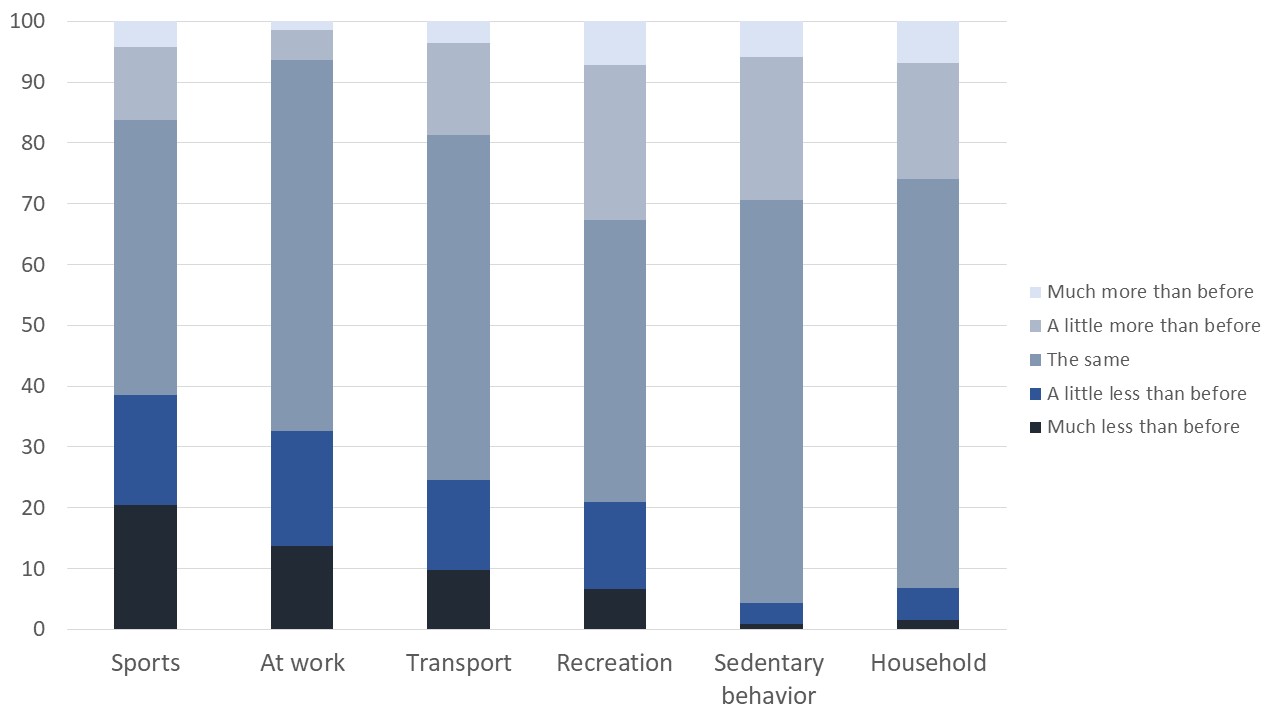


**Supplement 4. Interaction between age and the development of depressive symptoms on the change of physical activity and sedentary behavior at the onset of the COVID-19 restrictions**

|  | Sports | | | |  | At work | | | |  | Transport | | | |  | Recreation | | | |  | Sedentary behavior | | | |  | Household | | | |
| --- | --- | --- | --- | --- | --- | --- | --- | --- | --- | --- | --- | --- | --- | --- | --- | --- | --- | --- | --- | --- | --- | --- | --- | --- | --- | --- | --- | --- | --- |
|  | β | 95%-CI | | |  | β | 95%-CI | | |  | β | 95%-CI | | |  | β | 95%-CI | | |  | β | 95%-CI | | |  | β | 95%-CI | | |
|  |  |  |  |  |  |  |  |  |  |  |  |  |  |  |  |  |  |  |  |  |  |  |  |  |  |  |  |  |  |
| Age | -0.05 | (-.054 | -.042 | ) |  | 0.04 | (.037 | .048 | ) |  | 0.00 | (-.009 | .003 | ) |  | -0.03 | (-.033 | -.02 | ) |  | -0.07 | (-.073 | -.062 | ) |  | -0.08 | (-.081 | -.069 | ) |
| Δ depression (T2-T1) | -0.06 | (-.065 | -.053 | ) |  | -0.09 | (-.092 | -.08 | ) |  | -0.07 | (-.076 | -.064 | ) |  | -0.07 | (-.081 | -.069 | ) |  | 0.06 | (.058 | .07 | ) |  | -0.02 | (-.024 | -.012 | ) |
|  |  |  |  |  |  |  |  |  |  |  |  |  |  |  |  |  |  |  |  |  |  |  |  |  |  |  |  |  |  |
| Interaction | 0.00 | (-.006 | .004 | ) |  | 0.00 | (-.004 | .005 | ) |  | 0.00 | (-.001 | .009 | ) |  | 0.00 | (-.004 | .006 | ) |  | -0.02 | (-.027 | -.017 | ) |  | 0.00 | (-.008 | .002 | ) |
| Age * Δ depression (T2-T1) |  |  |  |  |  |  |  |  |  |  |  |  |  |  |  |  |  |  |  |  |  |  |  |  |  |  |  |  |  |
| Notes. *N* = 152,421; values of changes in sedentary behavior and all physical activity domains ranged from -2 to 2; additionally, adjusted for education, altered working conditions, self-rated health, anxiety, depression at baseline, living alone, feeling of loneliness, member of a sports club, physical activity at baseline, high regional number of COVID-19-cases, month of interview | | | | | | | | | | | | | | | | | | | | | | | | | | | | | |
